# Are lifestyle cardiovascular disease risk factors associated with pre-hypertension in 15–18 years rural Nigerian youth? A cross sectional study

**DOI:** 10.1186/s12872-015-0134-x

**Published:** 2015-11-04

**Authors:** N. A. Odunaiya, Q. A. Louw, K. A. Grimmer

**Affiliations:** Division of Physiotherapy, Stellenbosch University, Cape Town, South Africa; Department of Physiotherapy, University of Ibadan, Ibadan, Nigeria; International Center for Allied Health Evidence, University of South Australia, Adelaide, Australia

**Keywords:** Lifestyle CVD risk factors, Pre hypertension, Adolescents, Rural, Nigeria

## Abstract

**Background:**

Cardiovascular disease (CVD) is a public health concern worldwide. Hypertensive heart disease is predominant in Nigeria. To effectively reduce CVD in Nigeria, the prevalence of, and factors associated with, pre-hypertension in Nigerian youth first need to be established.

**Methods:**

A locally-validated CVD risk factor survey was completed by 15–18 year olds in a rural setting in south-west Nigeria. Body Mass Index (BMI), waist-hip ratio and systolic and diastolic blood pressure was measured. Putative risk factors were tested in gender-specific hypothesized causal pathways for overweight/obesity, and for pre-hypertension.

**Results:**

Of 1079 participants, prevalence of systolic pre-hypertension was 33.2 %, diastolic pre-hypertension prevalence approximated 5 %, and hypertension occurred in less than 10 % sample. There were no gender differences in prevalence of pre- hypertension, and significant predictors of systolic pre-hypertension (high BMI and older age) were identified. Considering high BMI, older age was a risk for both genders, whilst fried food preference was female-only risk, and low breakfast cereal intake was a male-only risk.

**Conclusion:**

Rural Nigerian adolescents are at-risk of future CVD because of lifestyle factors, and high prevalence of systolic pre-hypertension. Relevant interventions can now be proposed to reduce BMI and thus ameliorate future rural adult Nigerian CVD.

**Electronic supplementary material:**

The online version of this article (doi:10.1186/s12872-015-0134-x) contains supplementary material, which is available to authorized users.

## Background

The emerging epidemic of cardiovascular disease (CVD) in developing countries is a serious public health concern, particularly because CVD mortality in developing countries is higher in younger people than in developed countries [1, 2].

In Nigeria, CVD is fourth amongst the top 20 diseases responsible for mortality [3]. Hypertension, a precursor of hypertensive heart disease, is present in up to 46 % Nigerian adult population, and in an increasing percentage of Nigerian adolescents [4, 5]. Pre-hypertension has many complications including heart disease, stroke and kidney failure [6]. Hypertension can be linked with lifestyle risk factors such as smoking, harmful use of alcohol, low physical activity and high cholesterol diet, as well as non-modifiable risk factors such as advancing age and family history [7, 8].

Adolescent pre- hypertension is receiving increasing attention in the literature [9–11]. Adolescent pre-hypertension is a strong predictor of hypertension in adults [10], and progression from pre-hypertension to hypertension occurs faster, and at a younger age, in black people compared to Caucasians [12]. Consequently, investigating pre-hypertension in Nigerian adolescents is critical to inform effective interventions to reduce future adult CVD burden. Studies in developed countries have explored CVD risk factors and socio-economic factors associated with pre hypertension in urban adolescents [13–15]. However, only a few studies in Nigeria have investigated prevalence of hypertension and pre hypertension in urban adolescents [5, 16, 17], and no study has ascertained the prevalence of pre-hypertension and associated lifestyle risk factors among Nigerian rural adolescents. This paper reports on the first-known study in adolescents attending schools in rural Nigeria, of the prevalence of pre-hypertension and hypertension, and its associated lifestyle factors.

## Methods

### Ethics approval

Ethics approval was provided by Stellenbosch University Health Research Ethics Committee (No 8/09/257, 2009), and from the local educational authority in Ibarapa central local government area of Oyo State, Nigeria. School principals also gave permission, and presented the research to parent association meetings to facilitate informed consent and participation. Many adolescents in this community live with illiterate family members, thus verbal consent was accepted from parents/guardians. Written consent for each participant was also obtained from school principals, as in Nigerian culture they act as guardians to adolescents whilst in school.

### Sampling frame

#### Study setting

The study was conducted in 22 secondary schools in Ibarapa central local government. Twenty-three schools were initially identified through the local Inspectorate of Education Office, however during the research, one school closed. The Ibarapa central local government comprises two villages, Igboora and Idere. These have an estimated population of 57,000 (containing approximately 15,000 adolescents aged 10–19 years). It was estimated that 5400 of the population aged 15–18 years, would be attending secondary schools in the area at the time of the study.

#### Sample size calculation

An initial sample size of 1600 students was estimated, based on a cluster design effect of 5.8, an ICC of 0.5 to adjust for prevalence estimation and 90 % power for analyses. This calculation was based on the Nigerian adolescent CVD prevalence literature [5, 16, 17], and the estimated number of adolescents attending schools in the area. Sampling was originally planned in class clusters (i.e. whole classes selected would be invited to participate). Classes in the schools in this region generally contained adolescents of mixed age (10–20 years). However, based on information from school principals regarding the numbers of students likely to be absent from class on any day (truancy, illness, work etc), sampling was amended to a more practical approach, to invite all students aged 15–18 years in all participating schools. Thus the 1500 surveys that had already been printed would be distributed on a ‘first come first served’ basis, and an additional print was undertaken in case more surveys were required.

#### Sampling and data capture

All 15–18 year olds in the participating schools were invited to join the study, during an address at assembly in each school by the principal investigator. She met with interested adolescents and surveys were distributed. These were collected immediately after completion. The 1500 initially printed surveys were exhausted, and no further students came forward to participate, thus none of the additional surveys were required. Participants were present at school on the day of testing, agreed to complete the survey in class, had no obvious learning disabilities, and were able to complete the survey in either English or Yoruba language (the native local language).

### Study design

Cross sectional survey.

### Study measures

#### Lifestyle CVD risk factors

A specifically-developed and validated questionnaire for this rural Nigerian adolescent population measured lifestyle CVD risk factors by self-report [18]. The questionnaire used a 33 item profile scale with six ordinal or nominal subscales (demographics, CVD indicators, smoking, alcohol, physical activity and nutrition). The CVD indicator subscale identified family history of CVD, and was not used in this paper because adolescents may not have been aware of this information. The survey is included as Additional file 1.

After completing the survey, objective measurements of blood pressure (BP) and anthropometrics were taken by the principal researcher and two trained research assistants.

#### Body mass index (BMI)

Subjects wore light clothing without shoes. Weight was measured with a regularly-calibrated digital scale, and recorded to the nearest kilogram. Height was measured with a T-bar, and recorded to the nearest centimeter. BMI was calculated as weight (kgs)/height^2^ (metres).

#### Waist-hip ratio

Waist circumference was measured at the point of umbilicus, while hip circumference was measured at the widest point of the hip (both in centimetres). Waist-hip ratio was calculated as waist measurement divided by hip measurement.

#### Blood pressure

BP was measured with an automated sphygmomanometer. Acceptable psychometric properties of this instrument have been reported [19, 20]. This instrument has the advantage of eliminating the observer error found in the auscultatory method. BP was taken after ten minutes or more rest, following measurement of lifestyle factors, and anthropometrics. To be measured, adolescents sat on a chair with their arm resting on a table at heart level. The appropriate adolescent cuff size was used. BP was measured once on participants’ right arm. If BP was not within normal limits it was recorded again after a further 10 min rest and the average value used. We presumed that this might address any anxiety introduced by participating in the study. BP was then categorized as normal, pre-hypertensive or hypertensive ranges using African norms (see Table 1).

**Table 1:** Blood pressure cut off for Africa recommended by International forum for Hypertension in Africa for people 15 years and older (Lemogun et al, 2003) [21]

Normal blood pressure	Systolic <120 mm/Hg	Diastolic <80 mm/Hg
Pre-hypertension	Systolic = 80-89mm/Hg	Diastolic = 120–139mm/Hg
Hypertension	Systolic ≥ 140mm/Hg	Diastolic ≥90 mmHg
Stage 1 hypertension	Systolic = 140 -159mm/Hg	Diastolic = 90- 99mm/Hg
Stage 2 hypertension	Systolic = 160 -179mm/Hg	Diastolic ≥100

### Data management

Risk levels for study measures were defined as:

1. Smoking: any cigarettes/tobacco in the last 30 days
2. Alcohol: Any alcohol consumption
3. Low physical activity: less than 60 min exercise less than five days a week
4. High animal lipid diet: Eating meat (red meat, organ meat etc) five times per week or more
5. Low vegetable diet: Not eating vegetables at least five days a week
6. Low fruit diet: Not eating fruit at least five days per week
7. High salt diet- defined as adding additional salt to food
8. High BMI: Overweight was determined for those in the 85^th^ ≤ 95^th^ %, and obesity was determined for those ≥95^th^ [21, 22]. For interest, underweight was determined as those in the ≤5^th^% of the population distribution.
9. Abdominal obesity: There are no cut-off points for waist-hip ratio for Nigerian adolescents. Thus, the adult cut-offs were applied (waist-hip ratio: males ≥0.90; females ≥ 0.85 [23].

### Assessing associations

The CVD risk factors were initially regressed against themselves, to identify colinearity. Gender differences were identified in preliminary analysis in BMI, nutritional pattern, alcohol and tobacco intake, thus gender-specific causal pathways were determined *a priori*, using an interim fatness outcome measure (body mass index and waist-hip ratio), and a final outcome measure of systolic pre-hypertension. Predictor variables were tested for associations with the fatness indicators (comprising alcohol, low physical activity and poor dietary patterns). *A priori* directional associations were constructed for systolic pre-hypertension, with an interim outcome of fatness (and its associates of diet, alcohol and physical activity), as well as other variables potentially independently associated with systolic pre-hypertension (age, salt intake, tobacco, low physical activity). Composite gender-specific causal pathways were proposed, and tested using univariate and multivariate regression models.

### Statistical analysis

Data were descriptively reported using means and standard deviations, or percentages and 95 % confidence intervals (CIs) as appropriate. Univariate logistic regression models tested individual causal models, with findings reported as odds ratios (ORs) and 95 % CIs. Gender-specific step-wise multivariate models were constructed for systolic pre-hypertension, using high BMI as the primary exposure and taking into account other significant predictors from the univariate models, according to *a priori* reasoning, and the strength of association with systolic pre-hypertension. The value of adding each subsequent predictor variable to the model was determined by the amount of change in the likelihood ratio. A significant influence of the new variable exerted a change in likelihood ratio larger than the critical chi-square value associated with the degrees of freedom [24]. Moreover, potential confounders were identified as a change of 10 % or greater in the primary association between high BMI and systolic pre-hypertension [25]. All analyses were undertaken using SAS Version 9.2.

## Results

**Fig. 1:**
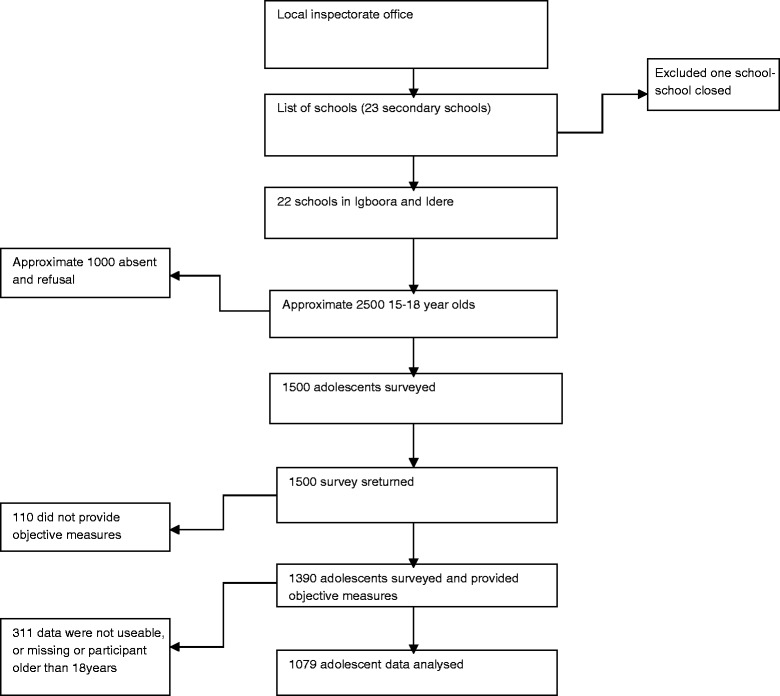
Study consort diagram

There were 1079 participants whose data could be analysed. Responses to invitation, participation in the study and data integrity are reported in Fig. 1.

### Sample descriptors

The sample comprised 53.5 % females and 46.55 % males. Overall, there was a high prevalence of systolic pre-hypertension (33.2 %), with much lower diastolic pre-hypertension prevalence (5.5 %). Systolic hypertension was 4.4 % overall, and diastolic hypertension was 0.8 %. Table 2 reports on gender-specific participant characteristics, as well as classifications of fatness, pre-hypertension and hypertension.

**Table 2:** Participant characteristics

Physical characteristics	Boys mean (SD)	Girls mean (SD)	Gender differences *(p value)*
Age (years)	16.4 (1.1)	16.3 (1.2)	*p* > 0.05
Height (cm)^a^	160.7 (9.4)	158.3 (6.1)	*p* < 0.05
Weight (kgs)	48.8 (8.3)	49.5 (7.3)	*p* > 0.05
Waist circumference (cm)^a^	26.3 (2.0)	25.5 (1.7)	*P* < 0.05
Hip circumference (cm)^a^	30.8 (2.5)	33.0 (2.6)	*p* < 0.05
Waist-hip ratio	0.8 (0.5)	0.8 (0.6)	*p* > 0.05
BMI^a^	18.8 (2.6)	19.7 (2.4)	*p* < 0.05
• Underweight %	9.6	9.7	*p* < 0.05
• Normal weight%	75.5	75.1	*p* < 0.05
• Overweight %	3.6	10.6	*p* < 0.05
• Obese %	11.4	4.7	*p* < 0.05
Systolic BP	116.6 (12.3)	115.4 (11.2)	*p* > 0.05
• pre-hypertension %	31.4	34.9	*p* > 0.05
• hypertension %	5.7	3.2	*p* > 0.05
Diastolic BP^a^	64.4 (8.5)	66.5 (8.9)	*P* < 0.05
• pre-hypertension %	4.2	6.7	*p* > 0.05
• hypertension %	0.8	0.9	*p* > 0.05

*Key* ^a^indicates significant differences

Modifiable CVD risk factors are described in Table 3. Prevalence of dietary risk factors was high for both males and females. Prevalence of any smoking and alcohol consumption were significantly higher in males than females, whilst females had significantly lower physical activity than males.

**Table 3:** Prevalence (%) of modifiable CVD risk factors among adolescents by gender (^a^significant gender differences)

CVD risk factors	Male (%) (95 % CI)	Female (%) 95 % CI	Total sample % (95 %CI)
Smoking/tobacco^a^	10.2 (7.5–12.8)	4.5 (2.8–6.2)	7.1 (5.6–8.7)
Alcohol use^a^	16.3 (13.1–19.6)	4.9 (3.1–6.60	10.8 (8.4–12.0)
Low fruit diet	10.4 (7.7–13.0)	6.8 (4.7–8.8)	42.2 (39.0–44.0)
Low vegetable diet	6.8 (4.6–8.9)	5.4 (3.5–7.2)	32.1 (29.5–35.1)
High salt diet	63.0 (58.8–67.2)	68.3 (64.5–72.1)	65.0 (62.0–67.0)
High animal lipid diet	61.2 (56.9–65.4)	58.2 (54.2–62.3)	59.8 (57.0–63.0)
Overweight^a^	15.0 (11.8–18.1)	15.2 (12.3–18.2)	15.2 (12.3–18.2)
Obesity^a^	1.8 (0.6–3.0)	5.4 (3.5–7.2)	5.7 (3.5–7.2)
High waist-hip ratio^a^	0.9 (0.6–1.3)	29.5 (17.6–36.6)	15.3 (13.2–17.5)
Low physical activity^a^	21.9 (18.3–25.5)	33.1 (29.3–27.0)	27.7 (25.2–30.6

*Key* ^a^indicates significant gender differences

### Association between modifiable CVD risk factors

There were significant gender-specific associations between CVD risk factors. High cholesterol diet was associated with low fruit consumption, low breakfast cereal, low vegetable consumption and low physical activity among females, whilst high cholesterol diet was associated with low fruit and low vegetable consumption (but not low breakfast cereal) among males. Alcohol and smoking were strongly associated for males and females (See Tables 4 and 5).

**Table 4:**
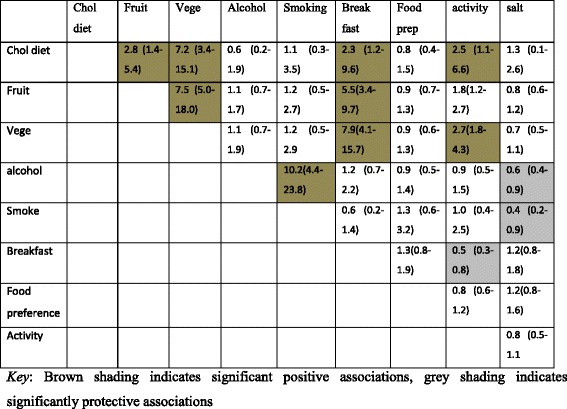
Associations among modifiable risk factors for females

**Table 5:**
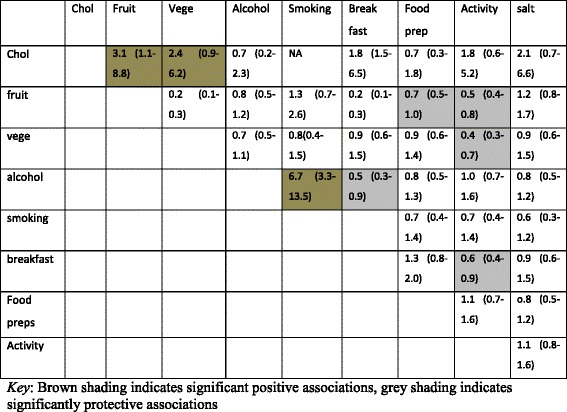
Associations among modifiable risk factors in males

### Association between BMI and waist hip ratio, and modifiable CVD risk factors

Considering high BMI (overweight and obesity combined), the association with older age was significant for males (17 or 18 years) and females (18 years) compared with younger age groups. Fried food preference was a significant predictor for females, while low intake of breakfast cereals was significant for males (See Table 6). Considering high waist-hip ratio, high cholesterol diet was protective only for males (OR 0.2 (0.04–0.9)). All other risk factors for this fatness measure showed no significant association. The non-significant findings are presented in Additional file 2, for interest.

**Table 6:**
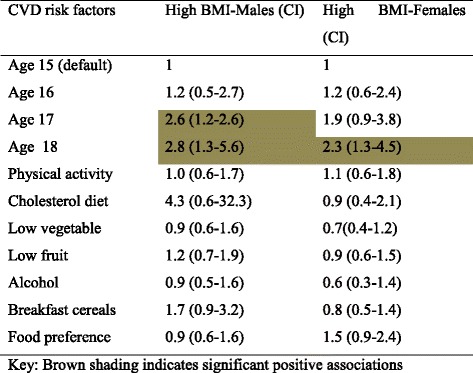
Associations between High BMI and individual CVD risk factors for males and females

### Associates of pre-hypertension

For males, three significant associates of systolic pre-hypertension were identified; aged 17 or 18 years compared to younger ages, high BMI (overweight and obesity) and any alcohol consumption. For diastolic pre-hypertension, high BMI was the only significant risk factor. For females, only one significant association was identified, for both systolic and diastolic pre-hypertension (high BMI). Findings are reported in Table 7.

**Table 7:**
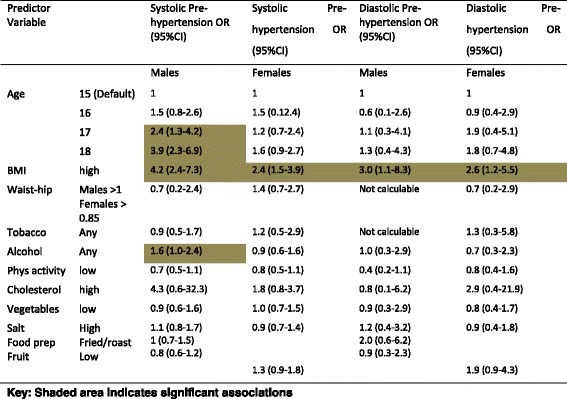
Gender-specific associates of age and modifiable CVD risk factors with pre-hypertension

During step-wise modelling to determine multiple associates for pre-hypertension, using the significant predictors identified from univariate analyses, only high BMI and age retained significant associations for males, whilst High BMI retained its significant association for females (See Table 8)

**Table 8:**
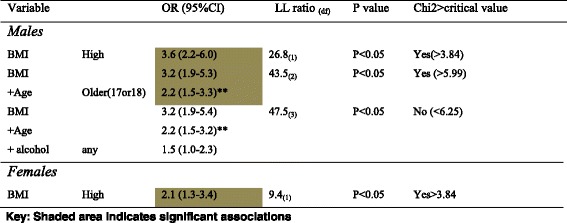
Stepwise regression modelling for systolic pre hypertension

.

## Discussion

This is the first known study in Nigeria investigating BP in rural adolescents, and associations between pre-hypertension and modifiable CVD risk factors. Systolic pre-hypertension was concerningly high for both male and female adolescents, and consistent associations were identified between systolic pre-hypertension, and high BMI and older-aged adolescents. The findings from this study should be applicable to adolescents living in other Nigerian rural districts because of the comprehensive sampling approach, and the large number of respondents. However a caveat is that it captured data only from students attending school, therefore pre-hypertension prevalence and CVD risk information for adolescents who attend school infrequently, or not at all, are not reflected here. The information from this study provides however, the beginnings of an evidence-base for planning effective intervention studies for at-risk rural Nigerian adolescent populations. Studies conducted in developed countries among adolescents have found similar associations between pre-hypertension, BMI and age [8–11], however the information from international studies could not be used to plan programs for adolescents in rural Nigeria for reasons such as local relevance, indigenous factors and other socio cultural influences on risk behaviors.

### Characteristics of participants

We recognise a gender-imbalance in our sample, probably related to adolescent females’ greater willingness to participate, and higher female enrollments and attendance in rural schools [26]. Personal communication from school principals suggested that adolescent males were more likely to truant from school, than females. There were also more fifteen years olds in the study than any other age group. This could be because younger adolescents were more likely to be attending school than older adolescents, and perhaps were more easily convinced to participate in the study than their older counterparts.

### Prevalence of pre-hypertension

The high prevalence of pre-hypertension in our sample reflects similar findings of other African studies. Pre hypertension among adolescents in semi-urban and urban southeast Nigeria was observed as 22.2 and 25 % respectively [17], whilst prevalence of pre hypertension in poor urban youth in Accra, Ghana, was reported as 32.3 % [27]. However the prevalence of pre hypertension in our study is higher than prevalence of pre hypertension observed in developed countries (15.7 %) [28] and 4.7 % Systolic BP,6.8 % Diastolic BP [29]. The high prevalence of pre-hypertension among our rural adolescents, and the similarity of our findings to other African studies, supports concerns about a pending epidemic of hypertension and CVD in rural Nigeria [4].

### Direction of association

We propose that in our study population, high BMI is an interim outcome of a directional association for pre-hypertension for both females and males, and that older adolescents are more at-risk of high BMI and subsequent pre-hypertension than younger adolescents. This being said, theorising appropriate causal pathways that integrated lifestyle factors and modifiable CVD risks with pre hypertension was challenging. For instance, associations between risk factors were different for adolescent males and females, and explaining these associations meant drawing on local knowledge and contexts. In both genders, alcohol was strongly associated with smoking. This relationship has been documented in other countries [30–33]. However this finding was new for rural Nigeria, as women are not expected to drink or smoke. It is generally believed in Nigerian communities, that women who drink are usually also involved in other social vices, thus such women may have significantly more at-risk behaviours, than the average man. This is not just a Nigerian finding. Morgen et al. [30] found elevated risk drinking and smoking among women, particularly in women with an early sexual debut. It is important for future studies into risk mitigation for pre-hypertensive Nigerian adolescent females to explore associated risk behaviors in order to make a real difference to future adult CVD.

### Dietary and activity findings

High cholesterol diet was associated with low fruit, low vegetable, low breakfast cereal and low physical activity in females, while for males, high cholesterol diet was associated with low fruit and low vegetable intake. This means that most poor dietary behaviours were linked. There is therefore, the need to educate Nigerian adolescents to eat balanced meals with lots of fruits and vegetables. However, high cholesterol diet was associated with high physical activity for males. This means that males with high cholesterol diets were likely to be more active than males with lower cholesterol diets. This requires more research to understand it.

Females on the other hand, who had poor dietary patterns, also had low physical activity levels. This is more readily explained. The high BMI finding for our adolescent females reflects a culture where African women are expected to be fat to show evidence of good care by their husbands. This belief is entrenched in a girl child especially in rural areas. Female physical activity (other than daily activities) is unusual, as a woman jogging in the street in a rural Nigerian setting could be perceived to be insane or being under a spell. Many religious practices do not encourage women to engage in sporting activities because it is not perceived to be feminine. Thus local contexts are essential when attempting to understand CVD risk factors, and how they could be addressed when planning prevention programs in Nigeria.

### Fatness and diet

For high BMI, fried food preference was associated for females and low breakfast cereal intake was associated for males. In a systematic review that included breakfast type, eating cereal was independently associated with lower BMI [34].

### Associations between pre-hypertension and CVD risk factors

We found that pre hypertension was independently associated with older adolescents (aged 17 and 18), compared to being aged 15 years. This concurs with adolescent studies conducted elsewhere (for instance China, Philippines, Ghana) [35–38], However, a study in the Congo [39] did not observe significant association between pre hypertension and age among adolescents. Apart from questions about study design and sampling which require critical appraisal of adolescent studies on this topic, this suggests that factors that determine adolescent pre-hypertension and hypertension may differ from place to place. This is another reason why the Nigerian rural context needs to be taken into account when planning effective interventions.

### Limitations of the study

#### Causality

The study was cross sectional, and thus we cannot infer causal relationships from the findings.

#### Artifacts in reading BP

The second measurement of BP was undertaken for adolescents with high first readings, to ensure that we truly were measuring high BP and not an artifact incurred from study participation anxiety. Taking the average of the two measures was our way of addressing this potential bias. We do not know however, whether the artifact remained in some participants in the second reading.

#### Biochemistry

We did not assess biochemical profiles of adolescents such as lipid profiles, thus our fatness measures are proxies for cholesterol.

## Conclusion

Our findings support a future CVD epidemic among adults in rural Nigeria because of the alarming prevalence of adolescent pre-hypertension. We suggest that rural Nigerian adolescents should be regularly screened for pre-hypertension and other lifestyle CVD risk factors, and educated about why screening, and intervention, is necessary. This paper provides evidence to support urgent development and implementation of culturally-appropriate CVD prevention programs in rural Nigeria. Since factors associated with BMI and pre hypertension were not the same for males and females, we propose that these CVD prevention programs should place gender-specific emphases on dietary habits, smoking and alcohol consumption, and physical activities. There is a need to further explore the influences of psychosocial, socioeconomic, familial and cultural factors on adolescent CVD risk factors in order to further fine-tune screening and intervention programs.

## Additional files

Additional file 1:
**Nigeria composite cvd risk factors questionnaire for adolescents.** (DOC 121 kb) Additional file 2:
**Associations between High Waist hip ratio and each of CVD risk factors among males and females.** (DOCX 13 kb)
