# Supplementary material for: Are lifestyle cardiovascular disease risk factors associated with pre-hypertension in 15–18 years rural Nigerian youth? A cross sectional study
Source: BMC Cardiovasc Disord. 2015 Nov 4;15:144. doi: 10.1186/s12872-015-0134-x (PMC4632346; doi:10.1186/s12872-015-0134-x)
Supplement: Additional file 1: — Nigeria composite cvd risk factors questionnaire for adolescents. (DOC 121 kb) [file 12872_2015_134_MOESM1_ESM.doc]

**NIGERIA COMPOSITE CVD RISK FACTORS QUESTIONNAIRE**

**FOR ADOLESCENTS**

Thank you for agreeing to complete this questionnaire which is about health behaviours that can cause heart disease. We have developed it to get information from you about things you do that may affect your heart health.

The information we are getting from you will be used to develop programs that will help young people like you. There are no wrong and right answers, so answer the questions based on what you know.

The answers you give will be kept secret and will only be used for research purpose.

Kindly read every question thoroughly and tick the right box. Use a pencil so you can erase any answer you tick by mistake.

**INSTRUCTIONS FOR COMPLETING QUESTIONNAIRE**

- **Use HB pencil only**
- **Tick your answer in the appropriate box.**
- **Where required, complete on dotted line provided.**
- **If you change your answer, erase your old answer completely.**
- **Each section deals with a different aspects such as personal details, etc.,**

**SECTION 1**

**Personal information**

**1.** Gender: **Male Female**

**2.** Date of Birth: **…………(yyyy)/……(mm)/……(dd)**

**3.** How old are you ?

12 years old or younger

13 years old

14 years old

15 years old

16 years old

17 years old

18 years old or older

**4.** In what class are you?

JSS3 grade

SSS1 grade

SSS2 grade

SSS3 grade

Other grades

**SECTION 2**

**CVD indicators and family history of CVD**

**5**. Do you get tired easily even when you don’t do exercise? Yes No

**6**. Do you have chest pain when you do exercises such as

running, playing football, pounding? Yes No

**7**. Do you find it difficult to breathe after a little exercise Yes No

such as climbing stairs, walking about for 10 minutes?

**8**. Do either of your parents or close relation have heart disease? Yes No

**9**. Do any of your parents or close relation receive treatment from a

doctor for heart disease, hypertension or diabetes? Yes No

**SECTION 3**

**Tobacco usage**

**10.** Answer YES if you have never smoked and do not intent

to smoke in future (else answer NO)? Yes No

**11**. You have tried cigarette smoking, even one or two puffs? Yes No

**12.** How old were you when you smoked a whole cigarette for the first time?

I have never smoked a whole cigarette

8 years old or younger

9 or 10 years old

11 or 12 years old

13 or 14 years old

15 or 16 years old

17 years old

18 years old or older

**13**. You have tried smoking, but not smoked in the past 30 days Yes No

**14**. Do you intent smoking in the future? Yes No

**15**. During the past 30 days, on how many days did you smoke cigarettes?

0 days

1 or 2 days

3 to 5 days

6 to 9 days

10 to 19 days

20 to 29 days

All 30 days

**16**. During the past 30 days, on the days you smoked, how many cigarettes did you smoke

per day?

I did not smoke cigarettes during the past 30 days

Less than 1 cigarette per day

1 cigarette per day

2 to 5 cigarettes per day

6 to 10 cigarettes per day

11 to 20 cigarettes per day

More than 20 cigarettes per day

**17.** You have smoked cigarettes daily, that is, at least one cigarette

every day for 30 days? * Yes No

**18**. During the past 30 days, on how many days did you use **chewing tobacco, snuff or smoke raw tobacco?**

0 days

1 or 2 days

3 to 5 days

6 to 9 days

10 to 19 days

20 to 29 days

All 30 days

**SECTION 4 : Alcohol usage**

*The next 4 questions ask about drinking alcohol. This includes drinking beer, wine, and hot drink such as ogogoro. For these questions, drinking alcohol does NOT include drinking a few sips of wine for religious purposes.*

**19**. How often do you have a drink containing alcohol?

Never

Monthly

2 - 4 times a month

2 - 3 times a week

4 or more times a week

**20**. How old were you when you had your first drink of alcohol (more than a few sips)?

I have never had a drink of alcohol other than a few sips

8 years old or younger

9 or 10 years old

11 or 12 years old

13 or 14 years old

15 or 16 years old

17 years old or older

**21**. How many standard drinks of alcohol ( 1 standard drink is about 350 ml or equivalent of one bottle of small coke) do you have on a typical day when drinking?

1 or 2

3 or 4

5 or 6

7 to 9

10 or more

**22.** How often do you have six or more drinks on one occasion?

Never

Less than monthly

Monthly

Weekly

Daily or almost daily

**SECTION 5: Physical activity**

**23.** During the past 7 days, on how many days were you physically active for a total of **at**

**least 60 minutes per day**? (Add up all the time you spend in any kind of physical

activity that increases your heart rate and makes you breathe hard some of the time.)

0 days

1 day

2 days

3 days

4 days

5 days

6 days

7 days

**24.** Which of the following activities have you done during the past seven days?

0 1-2 3-4 5-6 7 or more

Skipping

Rowing, canoeing

Brisk walk

Bicycling

Jogging or running

Aerobics

Swimming

Baseball or soft ball

Football

Dance

Badminton

Volley ball

Table tennis

Lawn tennis

Basket ball

Soccer

Cricket

Gardening /farming

Others

**25**. On an average school day, how many hours do you watch TV?

I do not watch TV on an average school day

Less than 1 hour per day

1 hour per day

2 hours per day

3 hours per day

4 hours per day

5 or more hours per day

**26.** On an average school day, how many hours do you play video or computer games or use a computer or handset for something that is not school work? (including activities such as computer games, and the Internet.)

I do not play video or computer games or use a computer for something that is not

school work

Less than 1 hour per day

1 hour per day

2 hours per day

3 hours per day

4 hours per day

5 or more hours per day

**27**. Do you have Physical education (PE) practical in your school time table? Yes No

**28**. In an average week when you are in school, on how many days do you go to Physical

Education (PE) practical classes?

0 days

1 day

2 days

3 days

4 days

5 days

**29**. During the past 12 months, on how many sporting competition/inter house sport did you take part? (Example; football, sprint, relay)

0 teams

1 team

2 teams

3 or more teams

**SECTION 6: Nutritional information**

**See next page!**

30. How many times a week do you eat each food on this list below?

|  | **Days per Week** | | | | | | |
| --- | --- | --- | --- | --- | --- | --- | --- |
|  | **1** | **2** | **3** | **4** | **5** | **6** | **7** |
| **MEAT** | | | | | | | |
| Pork |  |  |  |  |  |  |  |
| Turkey |  |  |  |  |  |  |  |
| Liver |  |  |  |  |  |  |  |
| Kidney |  |  |  |  |  |  |  |
| Other organ meat (intestine, etc) |  |  |  |  |  |  |  |
| Meat pies/pastries |  |  |  |  |  |  |  |
| Ready meals |  |  |  |  |  |  |  |
| Beef (cow meat) |  |  |  |  |  |  |  |
| Goat |  |  |  |  |  |  |  |
| Chicken |  |  |  |  |  |  |  |
| **BREAKFAST CEREALS** | | | | | | | |
| Pap (from millet, guinea corn etc |  |  |  |  |  |  |  |
| Golden morn |  |  |  |  |  |  |  |
| Corn flakes |  |  |  |  |  |  |  |
| Any other brands |  |  |  |  |  |  |  |
| **VEGETABLES** | | | | | | | |
| Salad vegetables |  |  |  |  |  |  |  |
| Leafy green vegetables  (E.g. green, ugwu, waterleaf, ewedu) |  |  |  |  |  |  |  |
| Green peas |  |  |  |  |  |  |  |
| Carrot |  |  |  |  |  |  |  |
| Pepper |  |  |  |  |  |  |  |
| Baked beans |  |  |  |  |  |  |  |
| Beans |  |  |  |  |  |  |  |
| Soya beans |  |  |  |  |  |  |  |
| Other |  |  |  |  |  |  |  |
| **FRUITS** | | | | | | | |
| Garden eggs |  |  |  |  |  |  |  |
| Oranges |  |  |  |  |  |  |  |
| Bananas |  |  |  |  |  |  |  |
| Grape fruits |  |  |  |  |  |  |  |
| Pawpaw |  |  |  |  |  |  |  |
| Mangoes |  |  |  |  |  |  |  |
| Cashew |  |  |  |  |  |  |  |
| Apples |  |  |  |  |  |  |  |
| Other |  |  |  |  |  |  |  |
| **Other FOOD** |  |  |  |  |  |  |  |
| Cassava and cassava products |  |  |  |  |  |  |  |
| Yam and yam product |  |  |  |  |  |  |  |
| Plantain |  |  |  |  |  |  |  |
| Bread |  |  |  |  |  |  |  |
| Eggs |  |  |  |  |  |  |  |
| Fish |  |  |  |  |  |  |  |
| Ice cream |  |  |  |  |  |  |  |
| Rice |  |  |  |  |  |  |  |
| Pasta/Noodles/Spaghetti |  |  |  |  |  |  |  |

**31**. Do you add salt to food at table if salt was already added to food

during the cooking process ? Yes No

**32**. How much salt do you eat compared to other young people like you?

More salt

Less salt

Same amount of salt

**33**. How do you prefer your food cooked? (E.g meat, fish, egg, plantain)

Boiled

Fried

Roasted

**This is the end of the survey.**

**Thank you !!**
