# Supplementary material for: Are lifestyle cardiovascular disease risk factors associated with pre-hypertension in 15–18 years rural Nigerian youth? A cross sectional study
Source: BMC Cardiovasc Disord. 2015 Nov 4;15:144. doi: 10.1186/s12872-015-0134-x (PMC4632346; doi:10.1186/s12872-015-0134-x)
Supplement: Additional file 2: — Associations between High Waist hip ratio and each of CVD risk factors among males and females. (DOCX 13 kb) [file 12872_2015_134_MOESM2_ESM.docx]

**Additional file 2** Associations between High Waist hip ratio and each of CVD risk factors among males and females

| **CVD risk factors** | **High W/Hip boys (CI)** | **High W/Hip girls (CI)** |
| --- | --- | --- |
| Age 15 (default) | 1 | 1 |
| Age 16 | 0.9 (0.2-3.3) | 0.8 (0.3-1.9) |
| Age 17 | 0.5 (0.1-2.9) | 0.7 (0.3-1.7) |
| Age 18 | 0.2 (0.02-1.8) | 1.5 (0.7-3.4) |
| Physical activity | 0.9 (0.3-3.1) | 0.9 (0.4-1.7) |
| Cholesterol diet | 0.2 (0.04-0.9) | 1.7 (0.4-7.3) |
| Low vegetable | 0.9 (0.3-3.1) | 0.9(0.4-1.7) |
| Low fruit | 0.4 (0.1-1.4) | 0.6 (0.3-1.2) |
| Alcohol | 0.5 (0.1-2.4) | 1.0 (0.4-2.4) |
| Breakfast cereals | 0.1 (0.5-31.5) | 0.6 (0.3-1.2) |
| Food preference | 0.2 (0.2-1.7) | 1.1 (0.6-2.1) |
